# Down-Regulation of *KORRIGAN*-Like Endo-β-1,4-Glucanase Genes Impacts Carbon Partitioning, Mycorrhizal Colonization and Biomass Production in *Populus*

**DOI:** 10.3389/fpls.2016.01455

**Published:** 2016-10-04

**Authors:** Udaya C. Kalluri, Raja S. Payyavula, Jessy L. Labbé, Nancy Engle, Garima Bali, Sara S. Jawdy, Robert W. Sykes, Mark Davis, Arthur Ragauskas, Gerald A. Tuskan, Timothy J. Tschaplinski

**Affiliations:** ^1^BioEnergy Science Center and Biosciences Division, Oak Ridge National Laboratory, Oak RidgeTN, USA; ^2^BioEnergy Science Center, School of Chemistry and Biochemistry, Institute of Paper Science and Technology, Georgia Institute of Technology, AtlantaGA, USA; ^3^The Biosciences Center, National Renewable Energy Laboratory, GoldenCO, USA; ^4^Oak Ridge National Laboratory – Department of Chemical and Biomolecular Engineering and Department of Forestry, Wildlife and Fisheries, University of Tennessee, KnoxvilleTN, USA

**Keywords:** plant cell wall, endo-β-1, 4-glucanase, carbon partitioning and allocation, cellulose, biomass, mycorrhiza, microbe, *Populus*

## Abstract

A greater understanding of the genetic regulation of plant cell wall remodeling and the impact of modified cell walls on plant performance is important for the development of sustainable biofuel crops. Here, we studied the impact of down-regulating KORRIGAN-like cell wall biosynthesis genes, belonging to the endo-β-1,4-glucanase gene family, on *Populus* growth, metabolism and the ability to interact with symbiotic microbes. The reductions in cellulose content and lignin syringyl-to-guaiacyl unit ratio, and increase in cellulose crystallinity of cell walls of *PdKOR* RNAi plants corroborated the functional role of PdKOR in cell wall biosynthesis. Altered metabolism and reduced growth characteristics of RNAi plants revealed new implications on carbon allocation and partitioning. The distinctive metabolome phenotype comprised of a higher phenolic and salicylic acid content, and reduced lignin, shikimic acid and maleic acid content relative to control. Plant sustainability implications of modified cell walls on beneficial plant-microbe interactions were explored via co-culture with an ectomycorrhizal fungus, *Laccaria bicolor*. A significant increase in the mycorrhization rate was observed in transgenic plants, leading to measurable beneficial growth effects. These findings present new evidence for functional interconnectedness of cellulose biosynthesis pathway, metabolism and mycorrhizal association in plants, and further emphasize the consideration of the sustainability implications of plant trait improvement efforts.

## Introduction

Plant cell walls play integral roles in determining plant form and function. Changes in the composition and structural properties of cell walls can have significant implications on the developmental and physiological processes relevant to water transport (Chen et al., 2005; Liang et al., 2010; Zhu et al., 2010), abiotic and biotic stress responses (Zhao and Dixon, 2014), and plant fitness (Yang et al., 2007; Goubet et al., 2009; Park et al., 2010; Thévenin et al., 2011). The relationship between plant cell wall integrity and biotic stress response, especially to pathogenic microbes, is well documented (Cantu et al., 2008; Bellincampi et al., 2014; Miedes et al., 2014). Genetic modification of the biosynthesis pathways of the major cell wall components; cellulose (Caño-Delgado et al., 2003; Hernández-Blanco et al., 2007), lignin (Tronchet et al., 2010), hemicellulose (Vega-Sánchez et al., 2012), and pectin (Bethke et al., 2016), have been shown to result in altered plant response to pathogen attack.

The impact of modified cell walls on biotic interactions with non-pathogenic symbiotic microbes or microbiomes is less understood. Emerging evidence suggests that genetically modifying the lignin biosynthesis pathway in plants can significantly alter the composition of the associated microbiome (Beckers et al., 2016). The functional impacts of modified cellulose properties on beneficial plant-microbe inter actions have received far less attention. Here, we investigated the functional impact of down-regulating *AtKOR*/*KORRIGAN-like* cellulose biosynthesis pathway genes, *PdKOR1* and *PdKOR2*, on cell wall composition, growth, metabolism and beneficial microbial interactions of *Populus deltoides*.

KORRIGAN (KOR), an endo-1,4-*β*-glucanase (EGase) belonging to the glycosyl hydrolase family 9 (GH9) (Henrissat, 1991; Urbanowicz et al., 2007), has been shown to be associated with the Cellulose Synthase Complex, CSC (Vain et al., 2014; Mansoori et al., 2014; Worden et al., 2015; Zhang et al., 2016), and to be integral to the pathways controlling content, crystallinity and degree of polymerization of cellulose in Arabidopsis *(kor-1* and *kor-2* and *irx2*) (Szyjanowicz et al., 2004; Takahashi et al., 2009). Modification of endo-1,4-β-glucanase has been shown to alter the defense response to *Pseudomonas syringae* (López-Cruz et al., 2014) in *Arabidopsis*. Previous studies in *Populus* have studied the phenotypic impact of simultaneous RNAi targeting of several *KOR* paralogs. Such an approach led to a reduction in cellulose content and secondary wall thickness with no apparent impacts on plant morphology in *P. euramericana* (Yu et al., 2014), and a contrasting phenotype of higher cellulose crystallinity, curled leaves and reduced height study in *P. alba* x *grandidentata* RNAi plants (Maloney and Mansfield, 2010).

Driven by the hypothesis that genetic modification of host cell wall can have cascading and quantifiable phenotypic impacts on its secondary metabolome and interaction with beneficial microbes, we investigated the consequence of a cellulose biosynthesis pathway gene modification on *Populus* metabolism and its ability to interact with a well-characterized fungal symbiont, *Laccaria bicolor* (Martin et al., 2008). Our results reveal significant phenotypic impacts, beyond the altered cellulose properties, to carbon partitioning (primary vs. secondary metabolites) and allocation (leaves vs. stem) pathways, and a distinctive secondary metabolome phenotype in *PdKOR* RNAi plants. Considering the biological, ecological, and economic significance of plant-microbe interactions, our results further emphasize the need for holistic considerations of plant trait modifications in crop improvement efforts.

## Materials and Methods

### Phylogeny and Protein Sequence Alignment

Protein sequences of the 26 *Populus* GH9 were collected from Phytozome v9.1 *P. trichocarpa* (Tuskan et al., 2006). The *P. trichocarpa* (*Ptr*) sequences, with significant sequence similarity to *AtKOR*, were labeled *PtrKOR1-5*. Correspondingly, *P. deltoides* (*Pd*) sequences were designated *PdKOR1-5* and were used as a phylogenetic/sequence reference in the design of qRT-PCR primers and the analyses of *P. deltoides* libraries and constructs. Protein sequences from other species were collected from NCBI. A phylogenetic tree was developed based on the maximum likelihood method in the MEGA5 software program (Tamura et al., 2011). Bootstrap values were calculated from 500 independent bootstrap runs. Protein sequence alignment and percent sequence similarity/identity were generated using the GeneDoc program (Nicholas et al., 1997).

### qRT-PCR

RNA extraction, using the Spectrum Plant Total RNA Kit (Sigma), and cDNA synthesis and qRT-PCR assays, were performed according to methods described previously (Payyavula et al., 2014). Primers are listed in Supplementary Table S1. The efficiency of these primers for use in *P. deltoides* was verified by evaluating PCR products from serial dilutions of *P. deltoides* cDNA libraries. Specificity was confirmed by performing a melt curve analysis on qRT-PCR reaction products and verifying the expected band size (amplicon sizes ranged from 60 to 240 bp) on an agarose gel.

### Plant Transformation, Growth and Sampling

A ∼200-bp fragment from the gene-specific 3′UTR of the *PdKOR1* and *PdKOR2* genes from *P. deltoides* was cloned in the binary vector pAGSM552 in both sense and antisense orientations to form a hairpin loop with a chalcone synthase intron and expressed under the control of a Ubiquitin promoter. *Agrobacterium*-mediated plant transformation of the wild-type *P. deltoides* ‘WV94’ line was performed at the ArborGen LLC facility in Ridgeville, SC. The gene-specific RNAi constructs were each used to generate a minimum of 20 independent transformation events or lines. Five clonal replicates (i.e., ramets) for each *PdKOR* transgenic line, along with equal numbers of clonal replicates for empty vector transformed control plants, were propagated at Oak Ridge National Laboratory greenhouses and maintained at a constant day/night temperature of 25°C with 16 h photoperiod. In a preliminary study, the empty vector transformed control plants performed similar to the wild-type, non-transformed plants (data not shown). As a result, we used the averaged data from three independent empty-vector lines as the control in all subsequent experiments. All plants were initially propagated in small tubes (up to 50 cm in height) and then moved to 6-L pots. After ∼6 months of growth, plant height and stem diameter were measured and stem samples were collected and air dried for cell wall chemistry analyses. Initial phenotypic assessments included 14 and 9 independent RNAi lines for *PdKOR1* and *PdKOR2*, respectively, and three replicated phenotyping cycles. In a second study, two RT-PCR-confirmed independent lines were propagated from greenwood cuttings to generate four to five ramets each. Estimates of leaf area were then calculated for five individual leaves between leaf plastochron index (LPI)-1 through LPI-5 for each ramet by multiplying midrib length by maximum width. At harvest time, the leaf at LPI-6, its petioles, phloem (i.e., excised bark), xylem (scraping from the stem) and remaining stem were collected. The phloem samples were frozen with dry ice. The stem samples were air dried and the remaining tissues were frozen in liquid nitrogen and stored at -80°C. Root weight was measured after thorough washing and drying at 70°C.

### Fungal Inoculations

For co-culture studies, the ectomycorrhizal fungal strain, *L. bicolor* S238N, was established and maintained on Pachlewski’s medium (Pachlewski and Pachlewska, 1974). Mycelia were produced in a peat-vermiculite nutrient mixture, then placed in glass jars and grown in darkness for 2 months at 25°C., They were subsequently stored at 4°C just prior to host inoculation (Duponnois and Garbaye, 1991). Co-culture was performed in the greenhouse with eight replicate plants for each line, according to prior published methods (Labbe et al., 2011). Soil inoculation was carried out with a pure fungal culture mixed in peat-vermiculite at a concentration of 10% (1:9, v:v). Control (no inoculation) plants were established in autoclaved peat-vermiculite soil. The percentage of mycorrhizal colonization was determined under a stereomicroscope as previously described (Labbe et al., 2011). In brief, washed roots were cut into 1 cm segments, and for each root system, 100 apices were randomly examined and assessed as either mycorrhizal or non-mycorrhizal. Fresh weights of leaves, stems and roots were recorded for each of the eight replicates per line.

### Non-structural Carbohydrate Analysis

Soluble sugars were estimated as previously described (Payyavula et al., 2012), with minor modifications. Briefly, freeze-dried leaf and air-dried stem samples (20 mg) were each extracted three times with 80% ethanol at 80°C. The supernatants were then combined and re-extracted with 50 mg of activated charcoal. Next, the pigment-free extract was dried overnight at 50°C to eliminate ethanol, resuspended in either 800 or 250 μl of water (leaf or stem, respectively) and then employed in sucrose and glucose estimation assays using commercial kits (Sigma, St. Louis, MO, USA). Starch was estimated from the plant sample fraction leftover after ethanol extract (pellet remaining after soluble sugar extraction). The pellet was treated with 720 μl of 0.1 N NaOH at 50°C for 30 min and was neutralized in 900 μl of 0.1 N acetic acid. Pellet starch was digested using 1 U each of *α-amylase* (from *Aspergillus oryzae*, Sigma, St. Louis, MO, USA) and amyloglucosidase (from *A. niger*, Sigma), as described elsewhere (Chow and Landhäusser, 2004; Payyavula et al., 2011). Starch quantities were calculated from the glucose standard calibration curve.

### Cellulose Content, and Lignin Content and Syringyl-to-Guaiacyl Ratio

Cellulose content was estimated by the anthrone method, as previously described (Updegraff, 1969), with modifications for a 96-well plate format (Payyavula et al., 2014). Lignin and the syringyl-to-guaiacyl ratio (S/G ratio) were analyzed at the National Renewable Energy Laboratory in Golden, CO by pyrolysis molecular beam mass spectrometry (MBMS), as described elsewhere (Tuskan et al., 2006; Sykes et al., 2009). Briefly, 4 mg stem samples were pyrolyzed at 500°C in a quartz reactor using a Frontier py2020 auto-sampler. The pyrolysis vapors were then analyzed using a custom Extrel single-quadrupole molecular beam mass spectrometer.

### Structural Carbohydrate Analysis

After sugar and starch extraction, the pellet was dried and used for structural carbohydrate analysis. The extract-free sample (5 mg) was hydrolyzed with 50 μl of H_2_SO_4_ (72%, v:v) at 37°C for 1 h then diluted with 1.45 ml of water and autoclaved for 60 min. After cooling, an aliquot was neutralized with CaCO_3_ and the carbohydrate composition was then analyzed as described elsewhere (Yee et al., 2012) on a high performance liquid chromatograph (LaChrom Elite^®^ system, Hitachi High Technologies America, Inc.) equipped with a refractive index detector (model L-2490) and a UV–Vis detector (model L-2420). Individual sugars were quantified based on the standard curve developed for each compound.

### Cellulose Crystallinity and Molecular Weight Measurements

*Populus* stem samples were ground and extracted in a dichloromethane solvent with a Foss Soxtec unit (Soxtec^TM^2050). Lignin and hemicellulose were removed from the extractive-free samples and used in a ^13^C cross-polarization magic angle spinning (CP/MAS) nuclear magnetic resonance (NMR) cellulose analysis by acetic acid holocellulose pulping and pre-acid hydrolysis, following published methodologies (Foston et al., 2011). We isolated α-cellulose from the holocellulose sample with an alkaline hemicellulose extraction procedure, as according to published literature (Foston et al., 2011), and then subjected it to a gel permeation chromatography (GPC) cellulose analysis.

In preparation for a ^13^C-CPMAS NMR analysis, 4 mm cylindrical ceramic MAS rotors were filled with isolated α-cellulose. Solid-state NMR measurements of ^13^C were carried out in a Bruker Avance-400 spectrometer operating at 100.55 MHz for with a Bruker double-resonance MAS probe spinning at 10 kHz. CP/MAS experiments utilized a 5 μs (90°) proton pulse and a 1.5 ms contact pulse with a 4 s recycle delay and 4–8 K scans. All spectra were recorded on equilibrated moisture samples (∼35% water content). Crystallinity was determined via a 2-peak integration of the C4 crystalline carbon region (δ 85–92 ppm) over the integral of the entire C4 region (δ 80–92 ppm), as published earlier (Wickholm et al., 1998; Foston et al., 2011).

The number-average molecular weight (M_n_) and weight-average molecular weight (M_w_) were determined by GPC after tricarbanilation of cellulose. Isolated cellulose from each sample was derivatized with phenyl isocyanate, following a published procedure (Wood et al., 1986; Foston et al., 2011). Prior to GPC analysis, derivatized cellulose was dissolved in tetrahydrofuran (THF, 1 mg ml^-1^), then filtered through a 0.45 mm filter and placed in a 2 ml auto-sampler vial. The molecular weight distributions of the cellulose tricarbanilate samples were analyzed by an Agilent GPC Security 1200 system equipped with four Waters Styragel columns (HR1, HR2, HR4, HR5), an Agilent refractive index detector and an Agilent UV detector (270 nm) using THF as the mobile phase with injection volumes of 20 ml. A calibration curve was constructed based on eight narrow polystyrene standards ranging in molecular weight from 1.5 × 103 to 3.6 × 10^6^ g mol^-1^. Data collection and processing were performed using the Polymer Standards Service WinGPC Unity software (Build 6807). Molecular weights (M_n_ and M_w_) were calculated using software relative to the universal polystyrene calibration curve. Number-average degree of polymerization (DP_n_) and weight-average degree of polymerization (DP_w_) were both obtained by dividing M_n_ and M_w_ by 519 g mol^-1^, the molecular weight of the tricarbanilated cellulose repeat unit. The polydispersity index was calculated by dividing M_w_ by M_n_.

### Metabolite Profiling

In preparation for Gas Chromatography (GC)-Mass Spectrometry (MS) metabolite profiling, approximately 50–75 mg (fresh weight) of mature leaf, phloem and xylem tissues were each extracted in 2.5 ml of 80% ethanol. The extracts were then combined in a 1.0 ml aliquot and dried in a nitrogen stream. Sorbitol was added before extraction and used as an internal standard. Dried extracts were dissolved in acetonitrile, followed by trimethylsilylation (TMS) and a GC-MS analysis was performed after 2 days, as described elsewhere (Jung et al., 2009; Li et al., 2012; Payyavula et al., 2014).

Metabolites of interest to be quantified were identified by database matching with the Wiley Registry 10th Edition combined with NIST 2014 mass spectral database and a large user-created database (>2300 spectra) of mass spectral EI fragmentation patterns of TMS-derivatized compounds. Peaks were integrated and analyzed using a key selected ion, a characteristic relatively unique m/z fragment for each peak, rather than the total ion chromatogram, to minimize integrating co-eluting metabolites. The extracted peaks of known metabolites were scaled back up to the total ion current using predetermined scaling factors. Unidentified metabolites used the scaling factor for the internal standard (sorbitol) and were denoted by their retention time (RT; min) as well as key mass-to-charge (m/z) ratios. Peaks were quantified by area integration and the concentrations were normalized to the quantity of the internal standard recovered, volume of sample extracted, derivatized, and injected, as described elsewhere (Tschaplinski et al., 2012). Fold-change values of metabolite levels in leaf, phloem and xylem tissues of RNAi plants were generated relative to control tissues. Student’s *t*-test was applied to identify significantly increased or decreased metabolites (*p* < 0.05; *n* ≥ 3).

For phenolic colorimetric analysis, 5 mg of freeze-dried leaf sample was extracted with 1.5 ml of 80% EtOH using a Tissue Lyzer (Qiagen, Valencia, CA, USA) for 5 min at 30 Hz. After centrifuging, the supernatant was analyzed for phenolics, chlorophyll and carotenoids, the pellet for tannins. Total phenolics were estimated in a 96-well plate by the Folin–Ciocalteu (FC) method, as previously described (Singleton and Rossi, 1965; Payyavula et al., 2014). Tannins from the pellet were estimated as described elsewhere (Porter et al., 1985). Specifically, 200 μl of 80% EtOH and 600 μl of butanol:HCl (95:5, v:v) containing 25 μl of 2% ferric-ammonium-sulfate (Sigma) in 2N HCl were combined with the pellet and heated at 95°C for 20 min. The sample’s absorbance was then recorded at A_550-700_ nm.

## Results

### Phylogenetic and Expression Analyses of *AtKOR*-Like, GH9A Family Endo-β-1,4-Glucanase Genes in *Populus*

**FIGURE 1:**
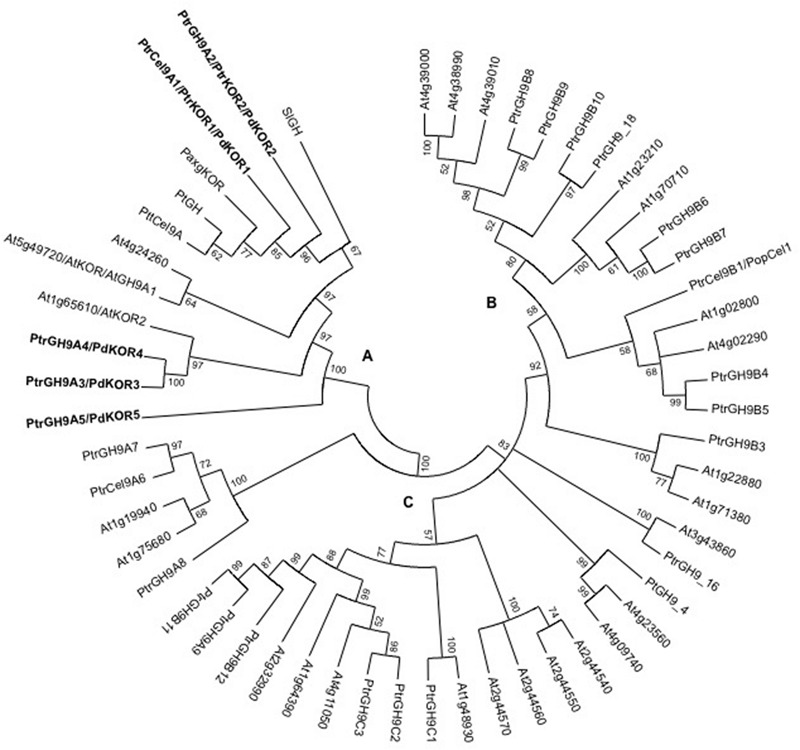
**Phylogenetic analyses of endo-β-1,4-glucanases from *Populus, Arabidopsis* and selected *KOR*-like genes from other species using MEGA5 program.** The evolutionary history was inferred by using the Maximum Likelihood method based on the JTT matrix-based model. The percentage of replicate trees in which the associated taxa clustered together in the bootstrap test (500 replicates) are shown next to the branches. The three distinct branches are represented by **(A–C)** subgroups. KORs from this study are in bold. The analysis involved 55 predicted protein sequences and the corresponding accession numbers are: At4g39000; At4g38990; At4g39010; At1g70710; At1g23210; At4g02290; At1g02800; At1g22880; At1g71380; At4g23560; At4g09740; At3g43860; At1g64390; At4g11050; At2g32990; At1g48930; At2g44570; At2g44560; At2g44540; At2g44550; At1g75680; At1g19940; At1g65610/AtKOR2; At5g49720/AtKOR/AtGH9A1; At4g24260; Pa*x*gKOR (*Populus alba* x *grandidentata*): ADB82903.1; PtGH (*P. tremuloides*): AAS45400.1); PtrKOR1/PdKOR1 (*P. trichocarpa*, Potri.003G151700); PtrKOR2/PdKOR2 (Potri.001G078900); PtrKOR3/PdKOR3 (Potri.008G079500); PtrKOR4/PdKOR4 (Potri.010G177300); PtrKOR5/PdKOR5 (Potri.005G188500); PtrCel9A6 (Potri.005G237700); PtrGH9A7 (Potri.002G023900);PtrGH9A8 (Potri.005G115400); PtrGH9A9 (Potri.002G225200); PtrCel9B1/PopCel1 (Potri.001G083200); PtrGH9B3 (Potri.019G069300); PtrGH9B4 (Poptri.014G126900); PtrGH9B5 (Potri.002G202400); PtrGH9B6 (Potri.010G109200); PtrGH9B7 (Potri.008G132700); PtrGH9B8 (Potri.009G123900); PtrGH9B9 (Potri.004G162200); PtrGH9B10 (Potri.015G12800); PtrGH9B11 (Potri.014G157600); PtrGH9B12 (Potri.001G356000); PtrGH9C1 (Potri.007G071200); PtrGH9C2 (Potri.003G139600); PtrGH9C3 (Potri.001G092200); PtGH9_4 (Potri.001G098800); PtrGH9_16 (Potri.006G219700); PtrGH9_18 (Potri.015G127900); PttCel9A (*P. tremula* x *tremuloides*):AAT75041.1); SlGH (*Solanum lycopersicum*): AAC49704.1

To understand the phylogenetic relationship and extent of sequence similarity among GH9 family members in *Populus*, we carried out sequence analyses as follows. Analysis of the amino acid sequences of all the annotated GH9 glycosyl hydrolase, endo -β-1,4-glucanase family members in the genomes of *P. trichocarpa* and *Arabidopsis* generated a tree with three distinct clades (**Figure 1**), designated, A, B, and C (Urbanowicz et al., 2007). Group A, which includes AtKOR, contains five *Populus* PdGH9A or PdKOR-like isoforms. At the amino acid level, PdKOR1 and PdKOR2 have a high degree of similarity with each other relative to the three other *Populus* paralogs in group A: PdKOR3, PdKOR4, and PdKOR5 (Supplementary Table S2). An analysis of the chromosomal positions showed that *PdKOR1* and *PdKOR2* lie within homeologous blocks (Supplementary Figure S1), which were created during the Salicoid whole-genome duplication event estimated to have occurred approximately 65 Mya (Tuskan et al., 2006; Kalluri et al., 2007).

**FIGURE 2:**
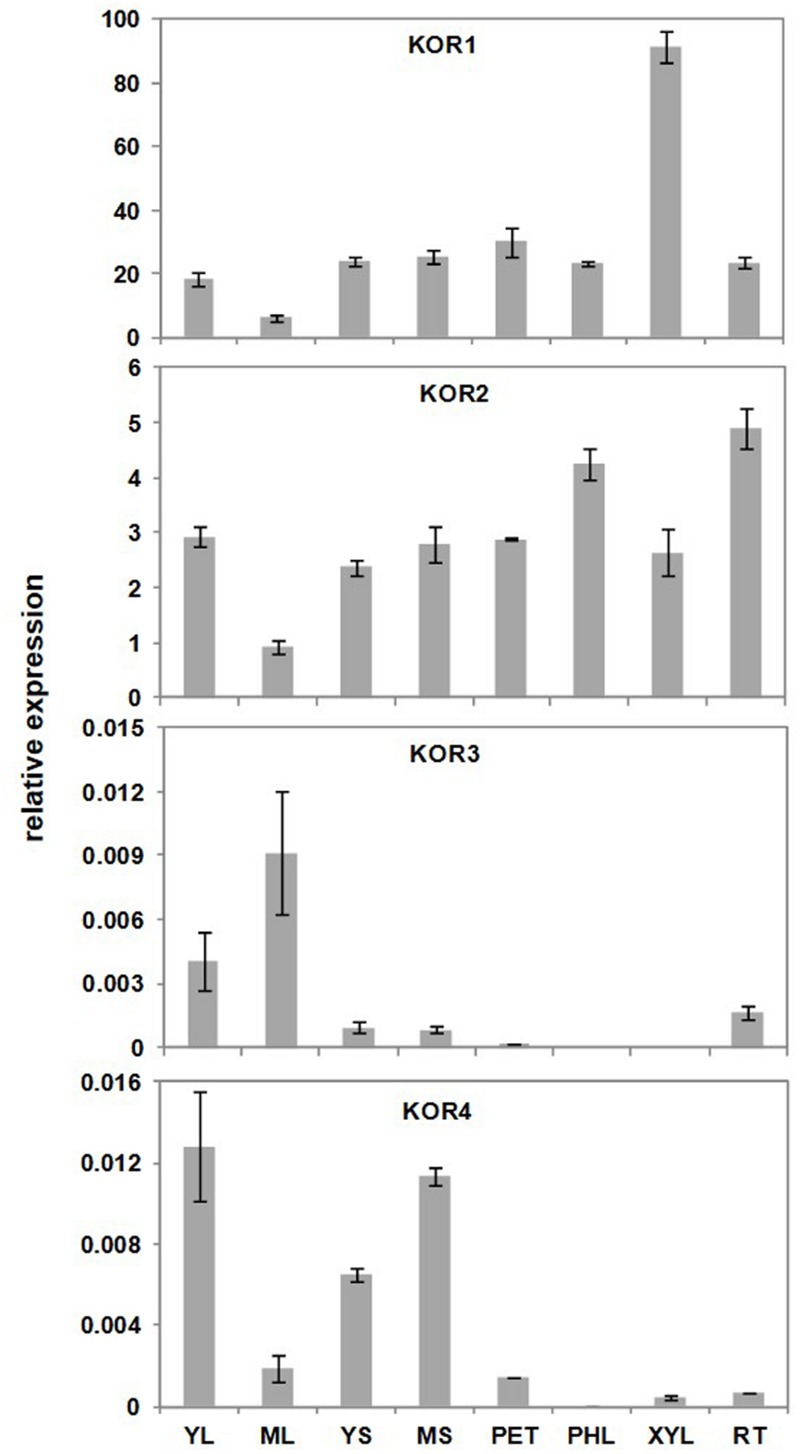
**Relative expression of *KOR-like* genes in various *Populus* tissue types.** Relative expression (arbitrary units) was based on difference in critical threshold (_C_T) values between target gene and average of housekeeping genes, *Ubiquitin-conjugating enzyme E2* and *18S ribosomal RNA*. Expression of *PdKOR5* (*KOR5*) was below quantifiable limits. YL, young leaf; ML, mature leaf; YS, young stem; MS, mature stem; PET, petiole; PHL, phloem; XYL, xylem; RT, root. Data represent means ± SE (*n* = 3).

Quantitative RT-PCR analyses showed that the relative expression level of *PdKOR1* gene was highest in xylem among various *Populus* tissue types measured (**Figure 2**). The expression level of *PdKOR2* gene were observed to be weaker across all tissues relative to *PdKOR1* gene. Expression levels of *PdKOR3* and *PdKOR4* genes were at significantly lower, at detection limit, in several tissues, while that of *PdKOR5* gene was below detection limit. An analysis of the cDNA sequences of the *PdKOR1, PdKOR2, PdKOR3, PdKOR4*, and *PdKOR5* paralogs in *Populus* showed the non-coding 3′ UTR region to be least conserved and hence this region was targeted in the design of gene-specific RNAi constructs (Supplementary Figures S2 and S3).

### Morphology and Growth of *PdKOR* Down-Regulated RNAi Plants

**FIGURE 3:**
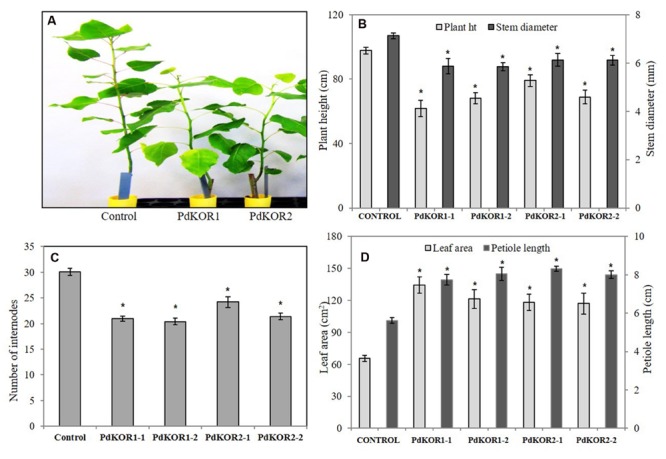
**Phenotypic characterization of *PdKOR1* RNAi, *PdKOR2* RNAi, and control plants. (A)** Image of a two-month old control and transgenic RNAi plants propagated from stem cuttings. Measurement of (B) Plant height and stem diameter, (C) Number of internodes, and (D) Leaf area and petiole length. Please refer to left-*y*-axis for plant height and leaf area, and to right-axis for stem girth and petiole length. Data represent mean ± SE (*n* = 4–5). ^∗^indicates statistically significant, *p* ≤ 0.05 based on Student’s *t*-tests.

*PdKOR1* and *PdKOR2* transgenic RNAi plants displayed a highly similar phenotype of slower growth and differential biomass accumulation relative to control plants. Independent lines from both *PdKOR1* and *PdKOR2* RNAi plants had reduced plant height, stem diameter and number of internodes, and larger leaves relative to control plants (**Figure 3**). A significant impact on growth and biomass properties of *PdKOR* RNAi plants was also observed. RNAi plants displayed a significant reduction in stem height and diameter, and number of internodes (**Figures 3A–C**), and an increase in leaf area and petiole length (**Figure 3D**).

We conducted RT-PCR analyses to study the specificity of the RNAi construct. RT-PCR assays showed a significant reduction in *PdKOR1* as well as *PdKOR2* transcript levels in either *PdKOR1* or *PdKOR2* RNAi background (Supplementary Figure S4A). The impact of RNAi down-regulation on transcript levels of *PdKOR3* and *PdKOR4* was found to be insignificant (Supplementary Figures S4B,C). *PdKOR5* transcripts were found to be below detection limit as in the case of native tissue expression profiling.

Given the down-regulation of both closely related *Populus* KOR paralogs, *PdKOR1* and *PdKOR2*, and similar phenotypes (**Figure 3**; Supplementary Figures S4A and S5), we henceforth refer to the transgenic RNAi lines as *PdKOR* lines and present results from representative lines of *PdKOR1*.

### Down-Regulation of *PdKOR* Alters Cellulose, Cell Wall and Biomass Properties of *Populus*

**FIGURE 4:**
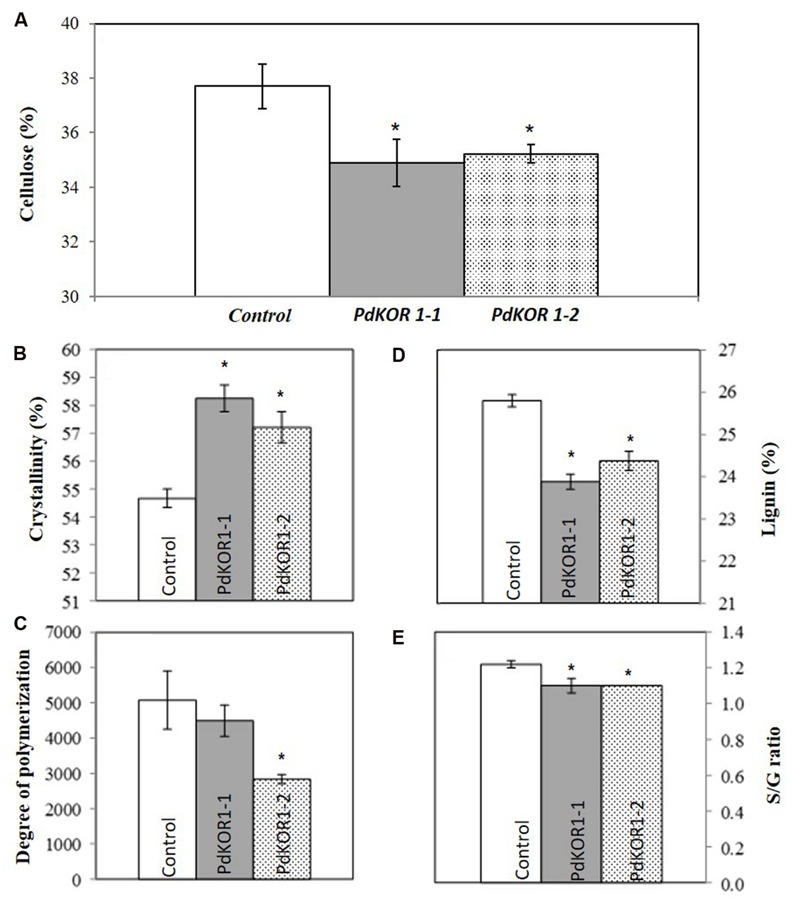
**Cell wall composition of control and *PdKOR1* downregulated lines. (A)** percentage (%) cellulose content, **(B)** NMR-based cellulose crystallinity, **(C)** degree of polymerization of cellulose, **(D)** lignin content, and **(E)** S/G (syringyl-to-guaiacyl) ratio are represented as means ± SE (*n* = 3–5). **(A–E)** represent values from debarked stem samples. ^∗^indicates statistically significant, *p* ≤ 0.05 based on Student’s *t*-tests.

In order to corroborate whether the sequence homologs of *AtKOR* have similar functional roles in cell wall biosynthesis in *P. deltoides*, we analyzed the cell walls of RNAi plants. *KOR* transgenic plants were characterized by reduced cellulose content (**Figure 4A**) and increased cellulose crystallinity (**Figure 4B**). Degree of polymerization (DP) measured by GPC technique yielded inconclusive results with a statistically significant reduction observed in only one of the *PdKOR RNAi* lines (**Figure 4C**). The effects of *PdKOR* down-regulation were measureable beyond cellulose to that of other cell wall properties. Statistically significant reductions in lignin content and S/G ratio were also evident (**Figures 4D,E**).

### Down-Regulation of *PdKOR* Impacts Carbon Metabolism, Carbon Partitioning and Allocation

The differential biomass observed for RNAi leaf, stem, and root relative to the control shows that carbon allocation is impacted in transgenic *PdKOR* plants. In order to understand the impact of modification of *KOR* genes on carbon partitioning, we complemented cell wall polymer analysis with analyses of soluble non-structural carbohydrates and phenolics.

**FIGURE 5:**
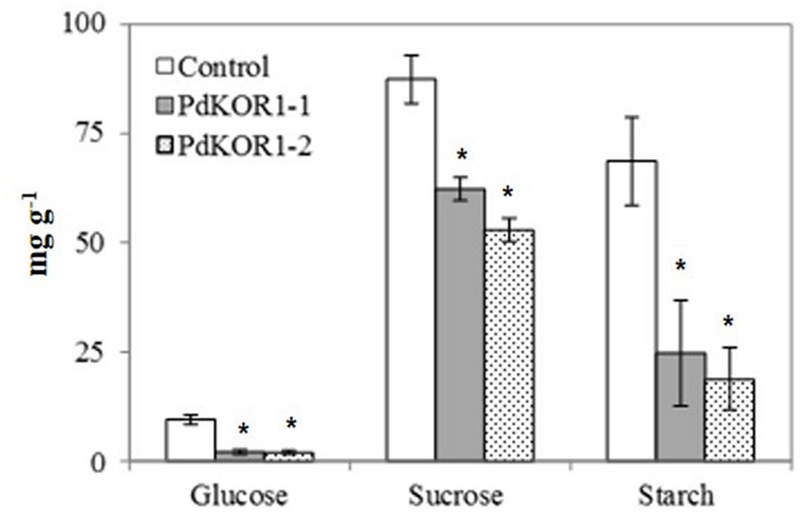
**Colorimetric estimation of non-structural carbohydrates in mature leaves.** Data are represented as means ± SE (*n* = 4). ^∗^indicates statistically significant, *p* ≤ 0.05 based on Student’s *t*-tests.

Our colorimetric analyses showed a relative decrease in the non-structural carbohydrate or sugar content in transgenic *PdKOR* RNAi plants. As such, the content of glucose, sucrose and starch in mature RNAi plant leaves was reduced by 30–80% relative to the control (**Figure 5**). In addition to confirming the colorimetric observation of reduced glucose content in RNAi plant tissues, MS analysis revealed a reduction in fructose, galactose and raffinose levels relative to the control.

We found that phenolic compounds were elevated in the RNAi plant leaves as well as phloem. Some of the predominantly phenolics were caffeic acid derivatives, including caffeoyl-glycosides, caffeoyl-shikimate conjugates, and other caffeoyl-conjugates (Supplementary Table S3). Two novel caffeoyl-glycoside conjugates, eluted at 14.14 and 19.31 min, were uniquely found in the *PdKOR* transgenic leaves, but not in the control. Syringin levels were reduced in nearly all of the lines. These changes in monolignol glucosides occur in parallel with an observed reduction of the S/G ratio in cell walls of RNAi plants. The observed reduction in carbon in the form of soluble sugars and increase in carbon represented as soluble phenolics suggests increased carbon partitioning to secondary metabolite pathways in RNAi plants (**Table 1** and Supplementary Table S4).

**Table 1:** Colorimetric estimation of total phenolics and tannins.

	Phenolics (mg g^-1^)	Tannins OD (g^-1^)
Control	128 ± 5	32 ± 3
PdKOR1-1	145 ± 5^∗^	21 ± 3^∗^
PdKOR1-2	146 ± 4^∗^	25 ± 2^∗^

*Data represent means ± SE (*n* = 4). ^∗^indicates statistically significant, *p* ≤ 0.05 based on Student’s *t*-test.*

The concentration of salicin, salicortin, and 6-hydroxy-2-cyclohexenone-1-carboxylic acid, a putative precursor of the latter, were elevated in all lines, coupled with a decline in 6-hydroxy-2-cyclohexenone (enol), 6-hydroxy-2-cyclohexenone alcohol and catechol, which are all putative breakdown products of salicortin. In the xylem of transgenic *PdKOR* lines, there was a significant decrease (>50%) in the concentrations of shikimic, maleic and threonic acid, and an increase (50–150%) in *cis-*aconitic acid (Supplementary Table S3).

### Down-Regulation of *PdKOR* Impacts Mycorrhizal Association with *Laccaria bicolor*

In order to test whether the specific genetic modification of the host cell wall and its cascading effects on secondary metabolome has a quantifiable phenotypic impact on the ability to interact with microbes, we grew both transgenic RNAi and control plants with and without the addition of the known beneficial fungal symbiont, *L. bicolor*, and observed the mycorrhizal association.

**FIGURE 6:**
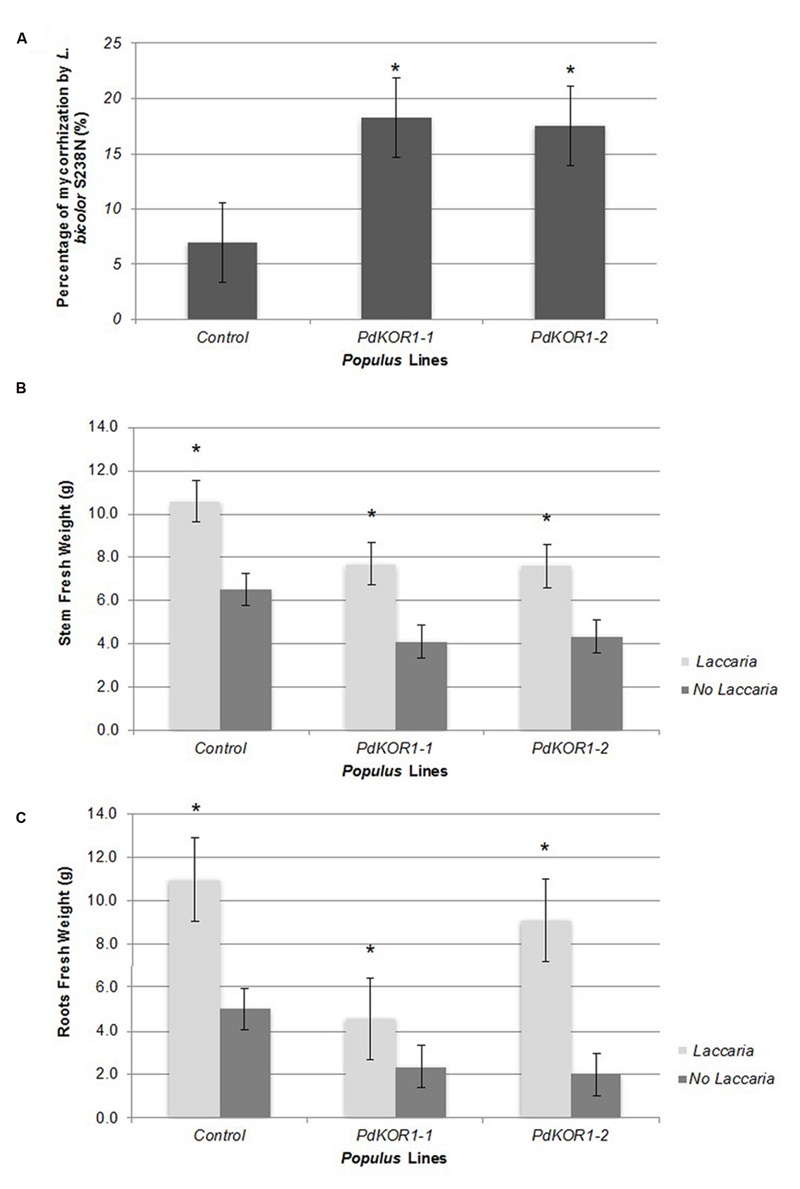
**(A)** Root colonization rate of the *Populus* transgenic *PdKOR1* downregulated and control lines by *Laccaria bicolor*. **(B)** Stem biomass of the plants with or without *L. bicolor* inoculation. **(C)** Root biomass weight of the *plants* with or without *L. bicolor* inoculation. ^∗^indicates statistically significant, *p* ≤ 0.05 based on Student’s *t*-tests.

*Laccaria bicolor* was found to have a higher colonization rate (i.e., mycorrhization rates) on the transgenic *PdKOR* plants relative to the control (**Figure 6A**). The greater mycorrhizal association also correlated with the significant gain in stem biomass in *PdKOR* RNAi lines relative to its non-inoculated plants following a two-month co-culture period in the greenhouse (**Figure 6B**). While an increase in root biomass was observed in inoculated vs. non-inoculated plants (**Figure 6C**), the gain in stem biomass in inoculated vs. non-inoculated plants was quantitative greater for *PdKOR* lines (74–90%) relative the control (61%) lines.

## Discussion

Delineating the genetic underpinnings and physiological implications of plant cell wall properties in bioenergy relevant crops such as *Populus* is critical to the goals of lignocellulosic bioenergy research. A cascading phenotypic effect of cellulose modification on carbon partitioning was distinctively supported by the metabolic profile of transgenic plants. The large diversity and fold-change ratios of higher-order caffeoyl-conjugates suggest that the reduced carbon flux to the lignin pathway resulted in a diversion of caffeic acid, via caffeoyl-quinates and caffeoyl-shikimates, to these higher-order caffeoyl-conjugates and may be symptomatic of dysfunctional cell wall assembly (Payyavula et al., 2014). The results suggest the phloem contains the large diversity of higher-order hydroxycinnamate-conjugates and is the organ that serves in both the storage and transfer of the carbon that was destined for lignin synthesis, but remained unassimilated. Several higher-order salicylates, including salicin and salicortin, are overrepresented in RNAi plants, owing to the diversion of carbon from structural lignin to these soluble phenolic microbial defense metabolites.

Previous studies using distinct species of *Populus* have showed that down-regulation of *PaxgKOR* (Maloney and Mansfield, 2010) and *KOR*-like gene from *P. euramericana* (Yu et al., 2014) resulted in modification in cellulose properties and a reduction of cell wall glucose content. Our *PdKOR* knockdown lines showed an increase in cellulose crystallinity, which is consistent with the results reported from down-regulation of *PaxgKOR*, (Maloney and Mansfield, 2010). However, this is contrary to the report of reduced crystallinity for *Arabidopsis* loss-of-function *irx2* mutants and *Arabidopsis* plants overexpressing a *Populus PttCel9A1* gene (Takahashi et al., 2009), as well as the report of unaffected crystallinity for *Picea KOR* (Maloney et al., 2012). Furthermore, the observed reduction in sucrose content of *PdKOR* RNAi leaves relative to the control is consistent with the results reported for *PaxgKOR* down-regulated plants (Maloney and Mansfield, 2010; Yu et al., 2014), however, the observed reduction in glucose levels is in contrast. It is important to note that the RNAi constructs in the two other studies are designed to target in conserved coding region, so as it is the case in this study both PdKOR1 and PdKOR2-like homologs were impacted. Taken together, these findings suggest that KOR’s role as a cellulose and cell wall modifying factor can differ qualitatively among species. The functional variations reported among studies may be attributable to differences in transgene technology applied, species-specific variations in protein isoforms and/or growth habit.

The enhanced mycorrhizal association, resulting in pronounced increases in co-culture induced root biomass, may be related to the differential metabolic and cell wall features of the *PdKOR* RNAi plants. The indirect effects on metabolome of RNAi plants potentially resulted in increased responsiveness, or priming of the root system, to its ectomycorrhizal partner (Tschaplinski et al., 2014). The altered lignin (Beckers et al., 2016), and shikimic (Tschaplinski et al., 2014), and phenolic and salicylic acid content (Lebeis et al., 2015), observed in *PdKOR* RNAi plants, have been previously reported as significant metabolites underlying plant-microbe signaling and interactions.

An alternate explanation for the enhanced mycorrhization may be drawn from the microbial biocontrol activity reported for cell wall degrading enzymes such as glucanases (Chet, 1987; Lam and Gaffney, 1993; Lorito et al., 1994; Lo, 1998; Harman, 2000). In this scenario, it may be speculated that PdKOR endo-β-1,4-glucanases have a direct functional role in defense and that down-regulation of the gene leads to a muted defense response phenotype, which promotes establishment of the plant-microbe relationship.

While there is evidence in literature for potential beneficial effects of mycorrhizae on plant growth and a suggestion of the importance of cell wall chemistry on mycorrhization (Goulao et al., 2011; Danielsen et al., 2013; Balestrini and Bonfante, 2014), a direct link to the beneficial effect of *Laccaria* on growth-compromised transgenic *Populus* plants and an underlying increase in mycorrhization rate has not been previously reported. It is plausible that increased nutrient acquisition, potentially nitrogen and phosphorus as a resulting from enhanced mycorrhization (Szuba, 2015) contributed to improved carbon assimilation and measurable growth benefits in transgenic *Populus* RNAi plants. These results provide an additional strategy to improve growth in transgenic plants that would otherwise produce below average biomass. The precise structural, molecular, and biochemical bases of improved mycorrhization rates, as well as the significance of the mutualistic relationship as a means of improving transgenic plant performance, merit further studies.

## Conclusion

The present study examined and validated the hypothesis that genetic modification of the host cell wall can have cascading and quantifiable phenotypic impacts on its secondary metabolome and its interaction with symbiotic microbes. It remains to be examined whether modifying endo-β-1,4-glucanase or a distinct cell wall pathway gene has an impact on functional microbiome associated with plants under field settings. It also remains to be clarified whether the pleiotropic phenotype is the result of a direct or an indirect, feed-back or feed-forward, cascade of changes triggered by the gene modification. Evaluation of additional plant mutants, genotypes or species, and plant metabolites for their interactions with microbes or microbiome are needed to delineate the specific structural and secondary metabolome characteristics influencing plant-microbe interactions.

## Author Contributions

UK, JL, and RP conceived the work, conducted analyses and wrote the manuscript. GT and TT conducted analyses and wrote the manuscript. RS, GB, TT, NE, SJ, AR, and MD, conducted analyses. UK and RP contributed equally to this work.

## Conflict of Interest Statement

The authors declare that the research was conducted in the absence of any commercial or financial relationships that could be construed as a potential conflict of interest.
