# Supplementary material for: Down-Regulation of KORRIGAN-Like Endo-β-1,4-Glucanase Genes Impacts Carbon Partitioning, Mycorrhizal Colonization and Biomass Production in Populus
Source: Front Plant Sci. 2016 Oct 4;7:1455. doi: 10.3389/fpls.2016.01455 (PMC5047894; doi:10.3389/fpls.2016.01455)

Supplementary Material

Down-regulation of endo-β-1,4-glucanase gene impacts carbon partitioning, mycorrhizal colonization and biomass production

Udaya C. Kalluri[1*], Raja S. Payyavula[1], Jessy L. Labbé[1], Nancy Engle[1], Garima Bali[2], Sara S. Jawdy[1], Robert W. Sykes[3], Mark Davis[3], Arthur Ragauskas[4], Gerald A. Tuskan[1] and Timothy J. Tschaplinski[1]

[1]Oak Ridge National Laboratory, BioEnergy Science Center and Biosciences Division, Oak Ridge, Tennessee, USA

[2]BioEnergy Science Center, School of Chemistry and Biochemistry, Institute of Paper Science and Technology, Georgia Institute of Technology, Atlanta, Georgia, USA

[3]National Renewable Energy Laboratory, The Biosciences Center, Golden, Colorado, USA

[4]Oak Ridge National Laboratory, University of Tennessee, Department of Chemical and Biomolecular Engineering and Department of Forestry, Wildlife and Fisheries, Knoxville, Tennessee, USA

[*]**Correspondence:**
Udaya C. Kalluri
kalluriudayc@ornl.gov

1 Supplementary Tables and Figures

1.1. Supplementary Tables

1.2. Supplementary Figures

1.1. Supplementary Tables

Table S1. Primer information.

List of primers and their sequences used in this study. RNAi primers were used for developing binary construct and the others were used to test gene expression.

Primer	Sequence
KOR1-F	TCGAAGCCAAACCCAAATAC
KOR1-R	TGCAACTAATCCTGCATTTCC
KOR2-F	AGCACTTGTGGCTTTGTCAG
KOR2-R	CTGTGGCATCTCATGGTCTC
KOR3-F	TCTCGAAATGGCTCGATACTTCC
KOR3-R	CAAGGTCAGCGCCTGAATTTAC
KOR4-F	CAACCATATTCTCGTGGGAAACC
KOR4-R	AGTGCCTTGGAGTGCTTAGA
KOR5-F	TCCAAGCAATAGTCCTGTCAAA
KOR5-R	GTCCAGCTCAAGAGGGTAATG
KOR1-RNAi-F	CACCCCCGGGGCTGCAATAGAGTTTGGACTT
KOR1-RNAi-R	TCTAGATACTAAGTATATCACAAAATCAGACAGA
KOR2-RNAi-F	CACCCCCGGGGTAAAGATAATGCGACTCCA
KOR2-RNAi-R	TCTAGACATATCATACACATATACTATTTATATCAA
18S F	AATTGTTGGTCTTCAACGAGGAA
18S R	AAAGGGCAGGGACGTAGTCAA
UBCc-F	CTGAAGAAGGAGATGACARCMCCA
UBCc-R	GCATCCCTTCAACACAGTTTCAMG

Table S2. Protein sequence similarity (percentage) matrix for selected KORRIGAN isoforms.

Full-length protein sequence similarity/identity % matrix for selected KORRIGAN-like isoforms. Accession numbers are as presented in Fig. 1.

	PdKOR2	PdKOR3	PdKOR4	PdKOR5	PaxgKOR	AtKOR	AtGH9A2	AtGH9A3	PgKOR
**PdKOR1**	96/94	72/59	60/49	53/38	98/97	90/82	71/55	84/73	88/78
**PdKOR2**		71/58	59/48	53/38	96/93	90/81	70/54	83/72	87/77
**PdKOR3**			74/69	53/37	72/59	72/59	75/60	68/55	73/58
**PdKOR4**				44/32	59/49	59/49	62/50	56/46	61/49
**PdKOR5**					52/37	51/37	50/34	50/36	52/37
**PaxgKOR**						90/82	71/54	83/73	88/78
**AtKOR**							71/54	85/77	86/74
**AtGH9A2**								70/53	71/53
**AtGH9A3**									80/68

Table S3. Fold-change of representative metabolites.

Fold-change of representative metabolite levels in leaf, phloem and xylem of RNAi plants compared to control. Values in red and blue indicate significantly increased or decreased, p<0.05, respectively, based on Student's t-tests. 'Inf' indicates present only in transgenic plants. Retention time (min) and mass to charge ratio for unknown compounds were given in parenthesis. Data represent means ± SE (n≥3).

compound	Leaf				Phloem			
	PdKOR1-1	PdKOR1-2	PdKOR2-1	PdKOR2-2	PdKOR1-1	PdKOR1-2	PdKOR2-1	PdKOR2-2
caffeoyl-glycoside-1	Inf	Inf	Inf	Inf	4.09	4.33	6.51	5.27
caffeoyl-glycoside-2	Inf	Inf	Inf	Inf				
caffeoyl-conjugate-1	9.90	10.89	13.84	8.78				
3-O-caffeoylquinic acid	3.61	4.89	4.87	3.42	1.58	1.35	1.61	1.16
unknown (17.5 min, 171, 219, 331)	3.43	6.18	8.78	4.55				
caffeoyl-shikimate-1	3.43	3.47	3.77	2.31	5.44	7.98	6.43	9.40
caffeoyl-glycoside-3	3.25	3.41	8.76	4.42	2.07	1.77	2.08	1.45
cis-3-O-caffeoylquinic acid	3.03	4.37	4.58	2.07				
caffeoyl-shikimate-2	2.95	3.71	7.09	3.29	6.32	7.88	8.91	10.50
citric acid	2.63	2.48	1.55	2.27	1.25	1.17	1.11	1.26
caffeoyl-shikimate-3	2.56	2.85	3.50	2.33	4.42	4.84	5.75	5.31
caffeoyl-conjugate-2	2.15	2.71	3.79	2.20	0.32	0.31	0.35	0.21
unknown (23.7min, 193, 271, 267, 355, 481, 571)	1.65	1.57	2.46	1.61	4.75	3.05	3.81	3.57
dihydroxybenzoic acid-galloyl-glycoside	1.33	2.00	2.04	1.95	1.19	1.52	1.27	1.42
catechol	1.31	1.42	1.51	1.63	0.60	0.57	0.64	0.60
salicylic acid	1.30	1.67	1.93	1.25	0.61	0.69	0.88	0.73
caffeic acid	1.13	1.20	1.99	0.82	1.22	1.31	1.67	1.47
salicortin breakdown product	1.09	1.54	2.28	1.29	0.47	0.41	0.51	0.45
unknown (23.53 min, 255, 193, 271, 481, 571)	1.09	0.98	1.49	1.24	1.73	1.88	1.85	1.48
salicyl alcohol	1.07	1.43	1.39	1.67	0.99	0.87	0.94	0.96
2,5-dihydroxybenzoic acid-5-O-glucoside	1.07	1.13	1.38	1.03	1.45	1.70	2.07	1.72
sucrose	1.01	1.05	0.97	0.87	1.10	1.05	1.10	1.12
salicyloyl-salicortin	1.00	1.27	1.45	1.82	0.58	0.58	0.69	0.66
unknown (9.66 min, 255)	0.85	0.93	1.23	1.15	2.39	2.06	2.32	2.60
a-salicyloylsalicin	0.82	1.32	2.36	1.06	0.48	0.69	0.70	0.70
glucose	0.79	0.79	0.71	0.81	0.68	0.57	0.62	0.49
unknown (23.17 min, 255, 193, 271, 481, 571)	0.75	0.69	0.96	1.04	1.46	1.41	1.42	1.20
salicin	0.75	1.07	1.56	1.03	1.49	1.72	1.87	1.92
coniferin	0.74	0.81	0.90	0.66	0.90	0.91	1.98	1.69
6-HCH (enol)	0.70	0.75	1.14	1.10	2.35	2.04	2.52	2.66
syringin	0.68	0.67	0.25	0.48	0.42	0.52	0.57	0.54
quinic acid	0.66	0.83	0.99	0.56	1.52	0.67	0.89	0.58
salicortin	0.60	0.96	1.44	1.00	0.50	0.55	0.39	0.60
unknown (11.16 min, 218)	0.55	0.55	0.49	0.53	0.77	0.65	0.62	0.71
glyceric acid	0.54	0.41	0.42	0.42	1.26	1.26	1.43	1.40
raffinose	0.54	0.34	0.31	0.30	0.46	0.51	0.56	0.40
caffeoylpopuloside	0.51	0.43	1.15	0.75	1.82	3.16	2.67	2.17
galactose	0.48	0.39	0.35	0.45	0.76	0.60	0.70	0.52
caffeoyl-conjugate-4	0.46	0.49	0.85	0.94	2.17	2.26	2.92	2.50
fructose	0.45	0.40	0.45	0.45	0.90	0.59	0.61	0.56
digalactosylglycerol	0.31	0.23	0.20	0.38	1.10	1.23	1.27	1.19
glycoside-2	0.24	0.40	0.28	0.19	0.76	0.79	0.79	0.73
caffeoyl-conjugate-5	0.20	0.28	0.24	0.18				
shikimic acid	0.16	0.18	0.42	0.21	0.75	0.53	0.53	0.47

Table S3. Fold-change of representative metabolites. (continued)

compound	Xylem			
	PdKOR1-1	PdKOR1-2	PdKOR2-1	PdKOR2-2
**putrescine**	6.70	3.21	1.59	1.38
**unknown (10.03 min, 159)**	3.65	6.12	2.86	2.73
**unknown (11.98 min, 232 449 359)**	2.34	5.72	2.42	2.40
**cis-aconitic acid**	2.19	2.54	1.47	1.86
**asparagine**	2.12	6.08	2.99	2.82
**oxo-proline**	1.99	2.13	1.26	1.39
**glycerol-1/3-phosphate**	1.96	1.58	1.49	1.39
**sucrose**	1.71	1.18	1.10	1.00
**unknown**	1.58	1.73	1.42	1.26
**GABA**	1.57	1.52	1.21	1.11
**glucose**	1.20	0.67	0.38	0.37
**galactose**	1.19	0.59	0.35	0.37
**salicin**	0.87	0.82	1.04	0.56
**raffinose**	0.82	0.69	0.59	0.28
**unknown (11.16 min, 306)**	0.52	0.39	0.40	0.33
**shikimic acid**	0.50	0.32	0.23	0.23
**threonic acid**	0.34	0.27	0.16	0.21
**maleic acid**	0.33	0.31	0.23	0.21
**fructose**	0.30	0.16	0.14	0.13

Table S4. Colorimetric estimation of total phenolics and tannins. Data represent means±SE (n=4). * indicates statistically significant, *p*≤0.05 based on Student's *t*-test.

	Phenolics (mg g^{-1})	Tannins OD (g^{-1})
**Control**	128 ± 5	32 ± 3
**PdKOR2-1**	172 ± 7*	30 ± 7
**PdKOR2-2**	155 ± 9*	24 ± 3*

1.2 Supplemental Figures

Figure S1. Chromosomal positions of the five five γ clade *PdKOR*-like genes.

Chromosomal positions of the five Group A, *PdGH9A*/*PdKOR*-like genes. Scale represents a 5 Mb chromosomal distance. Colors indicate the ancestral chromosome and chimeric nature of most linkage groups. Common colors refer to homeologous genome blocks, presumed to have arisen from the Salicoid genome duplication 65 Mya and shared by two chromosomes (Tuskan et al., 2006, Kalluri et al., 2007). Chromosome numbers (linkage group number I-XIX) and sizes (Mb) are indicated at the bottom end of each chromosome. *Populus* genomic loci representative of the five members of GH9 gene family subgroup A are indicated by black arrows.

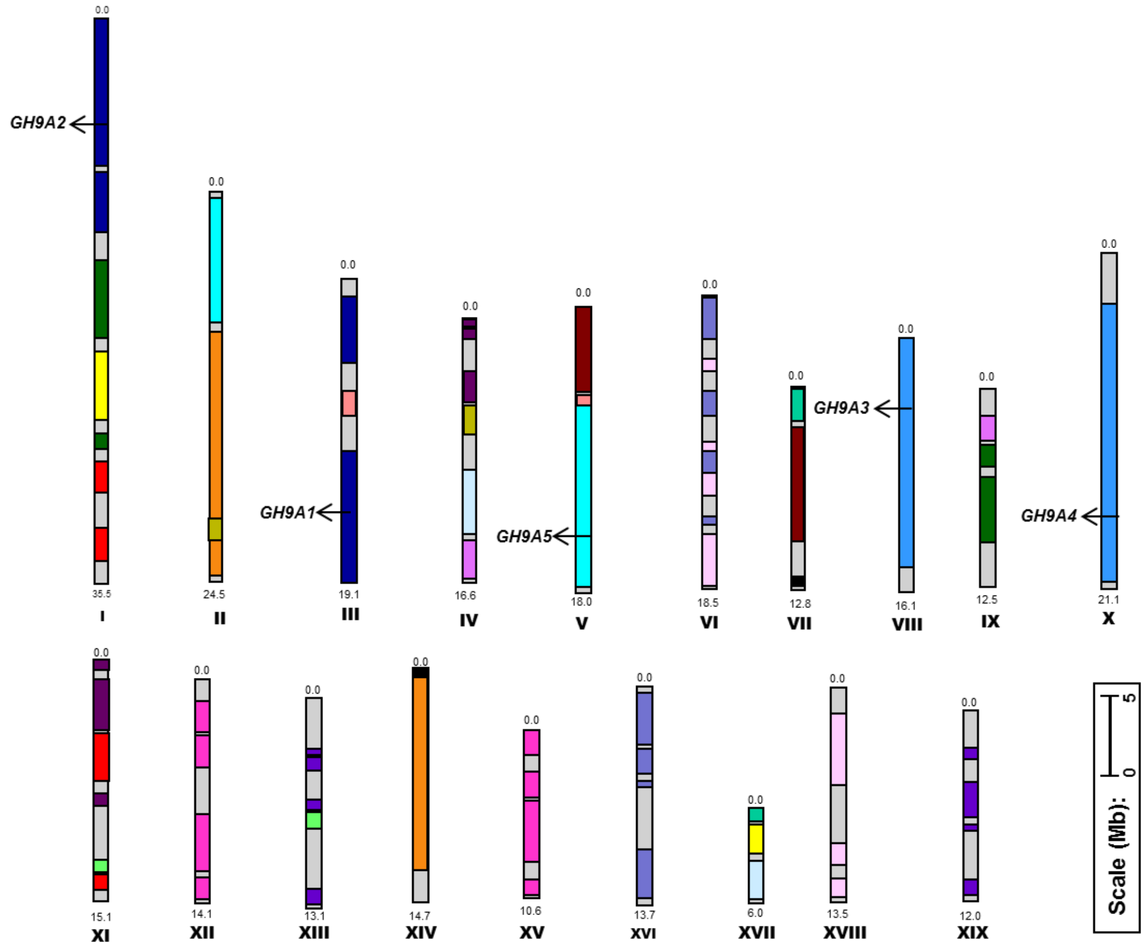

Figure S2. Sequence alignment of *PdKOR1* RNAi target sequence with cDNA sequences of *PdKOR1*, *PdKOR2, PdKOR3, PdKOR4* and *PdKOR5*. Consensus in sequences are highlighted with blue font color.

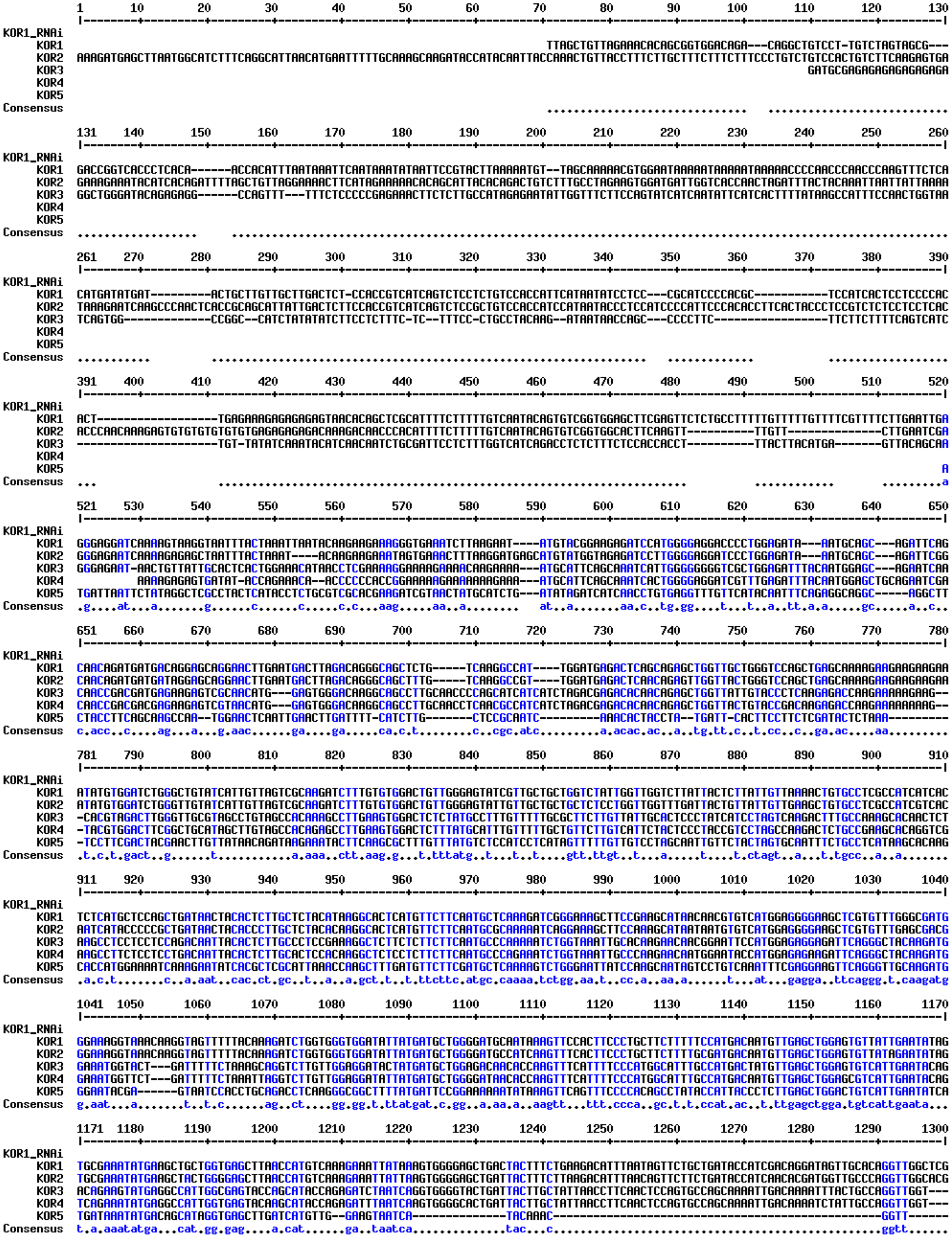

KOR1_RNAi
KOR1
KOR2
KOR3
KOR4
KOR5
Consensus
1 10 20 30 40 50 60 70 80 90 100 110 120 130

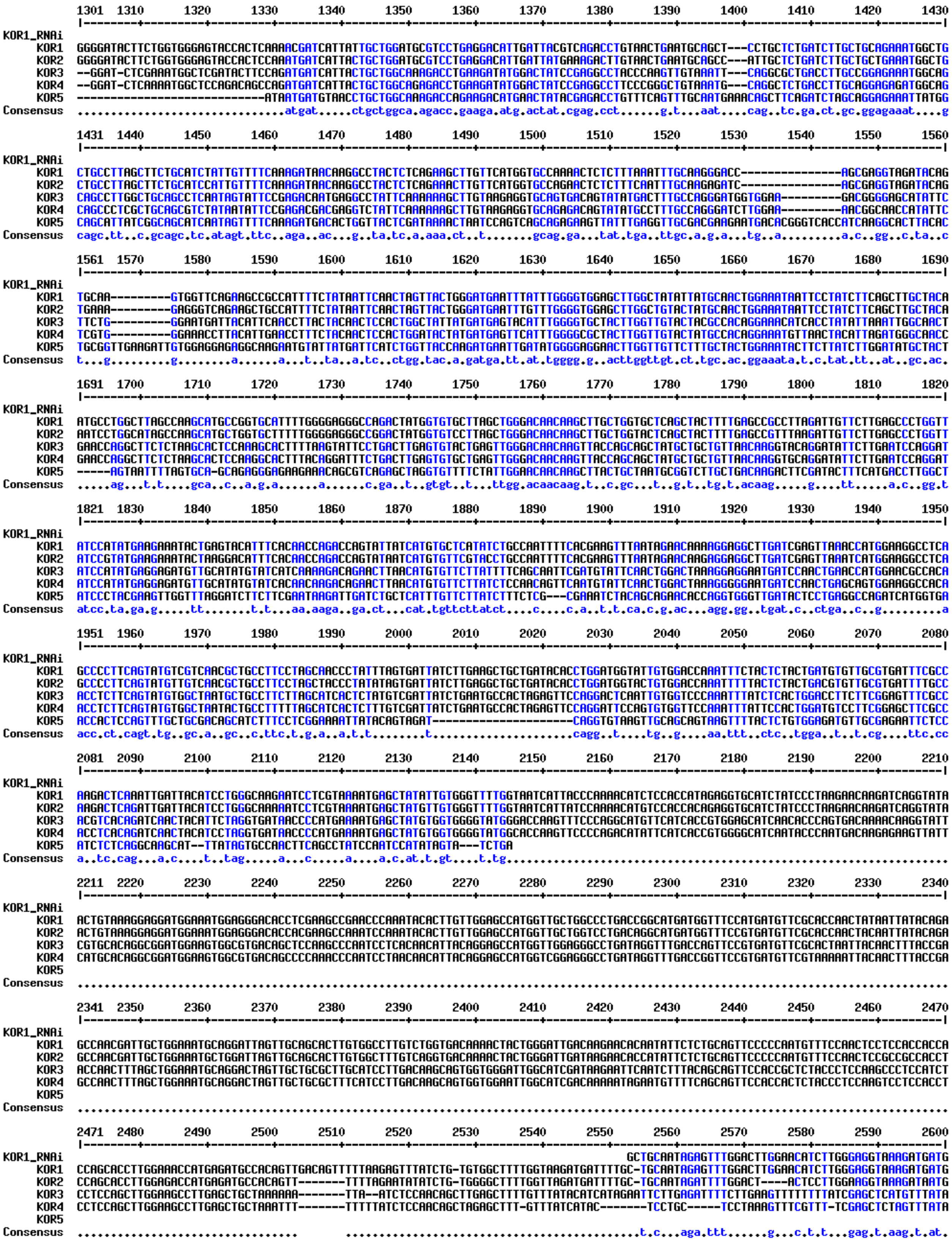

Figure S2 (Continued). Sequence alignment of *PdKOR1* RNAi target sequence

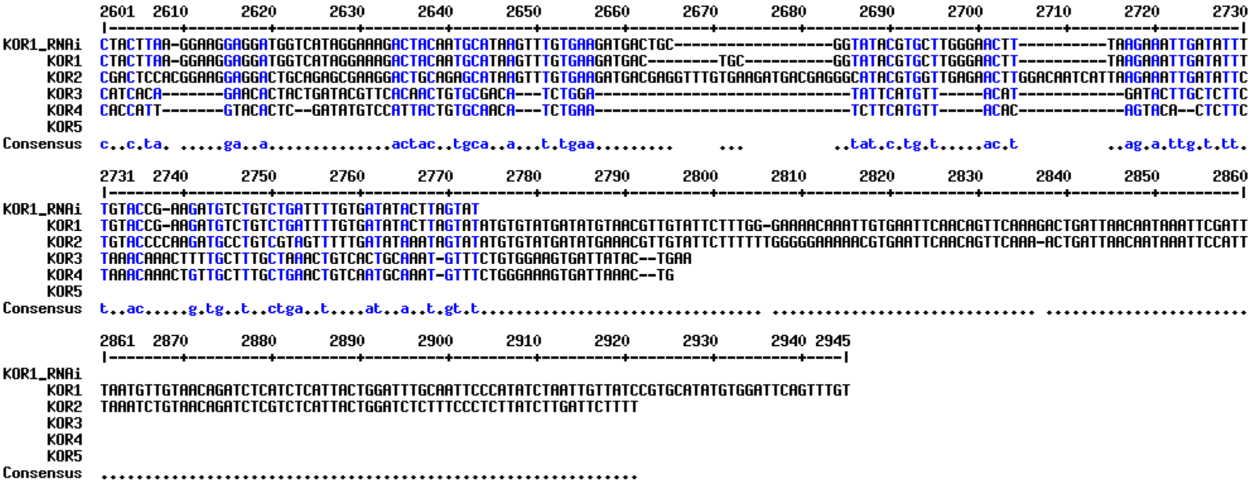

Figure S2 (Continued). Sequence alignment of *PdKOR1* RNAi target sequence

Figure S3. Sequence alignment of *PdKOR2* RNAi target sequence with cDNA sequences of *PdKOR1*, *PdKOR2*, *PdKOR3*, *PdKOR4* and *PdKOR5*. Consensus in sequences are highlighted with blue font color.

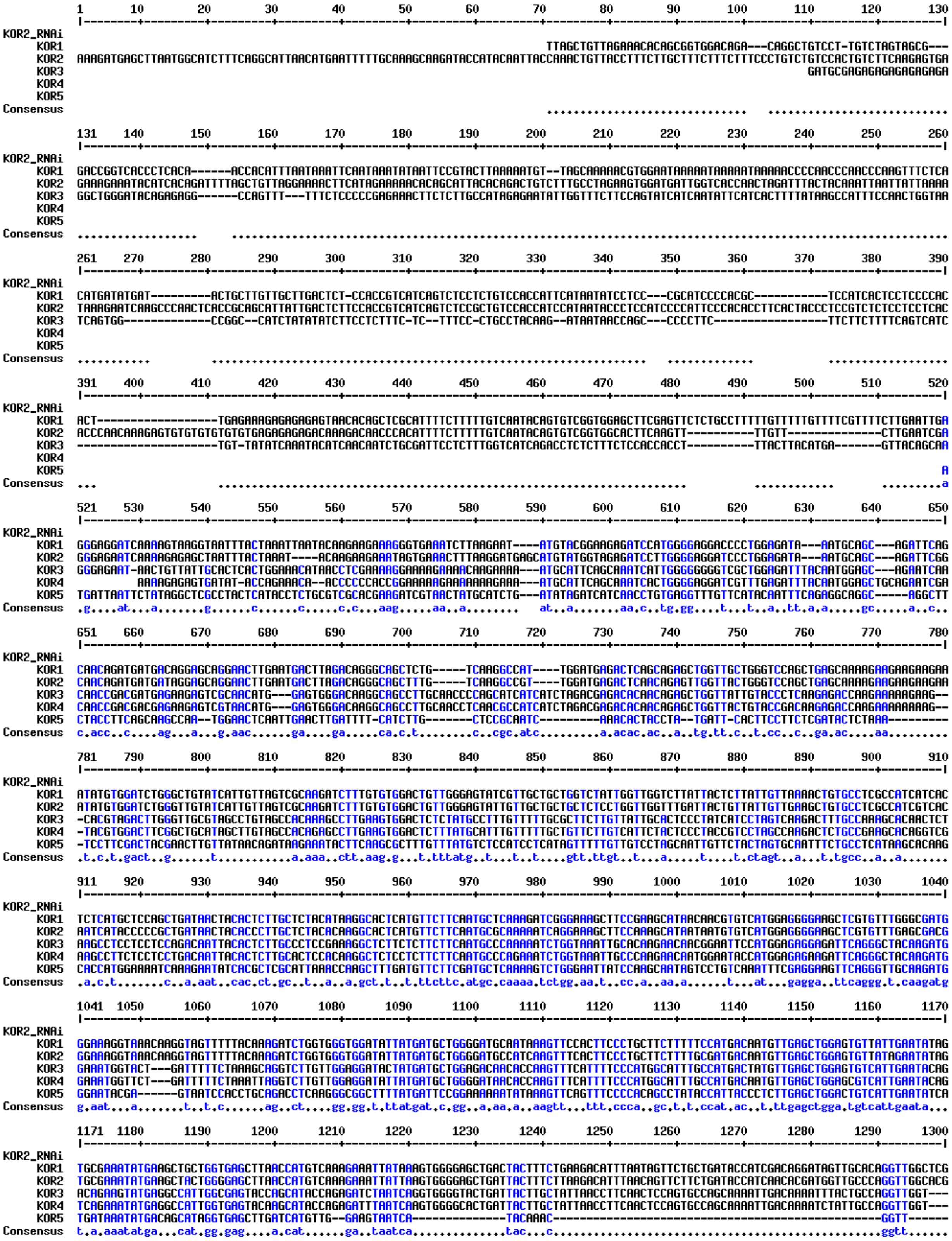
KOR2_RNAi
KOR1
KOR2
KOR3
KOR4
KOR5
Consensus

Figure S3. Sequence alignment of *PdKOR2* RNAi target sequence

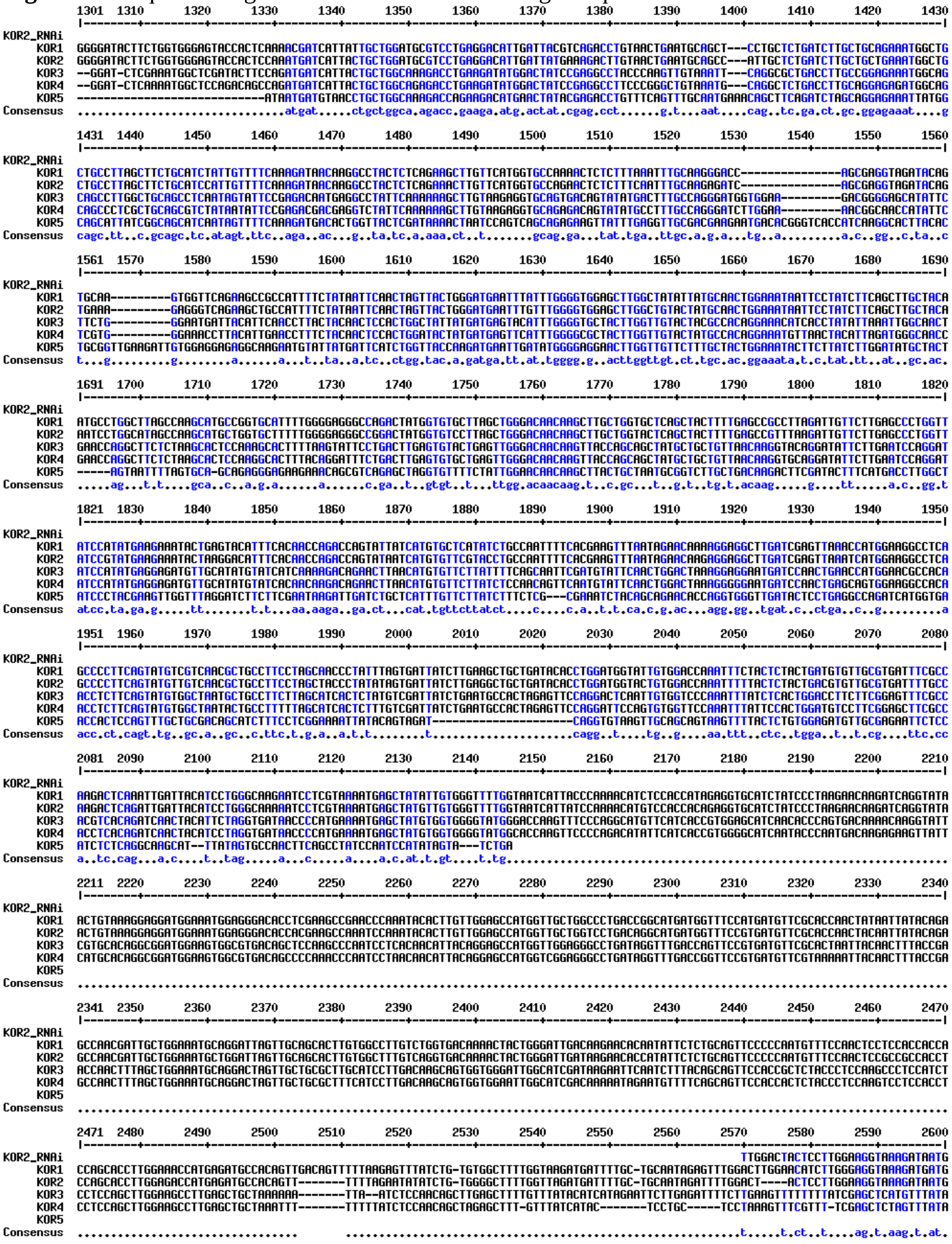

Figure S3 (Continued). Sequence alignment of *PdKOR2* RNAi target sequence

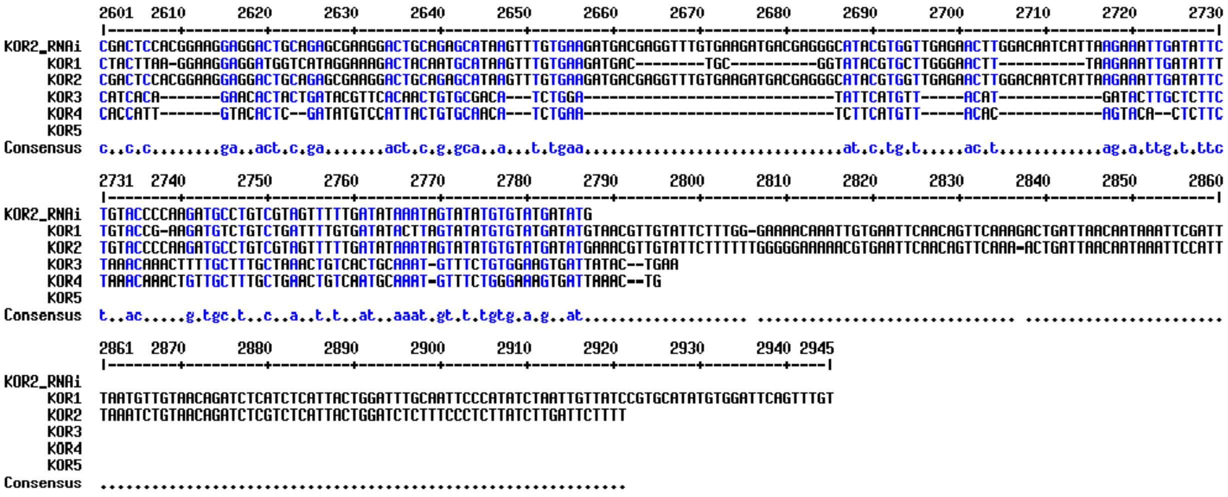

Figure S3 (Continued). Sequence alignment of *PdKOR2* RNAi target sequence

Figure S4. qRT-PCR confirmation of RNAi down-regulation.

Relative transcript abundance of (A) *PdKOR1* and *PdKOR2* and (B) *PdKOR3* and *PdKOR4* genes in control and transgenic lines. Relative expression was based on changes in critical threshold ($_C$T) values relative to housekeeping genes. (C) non-normalized expression values for *PdKOR3* and *PdKOR4* genes show their weak, near detection limit expression levels. Data represent means ± SE (n=3).

(A)

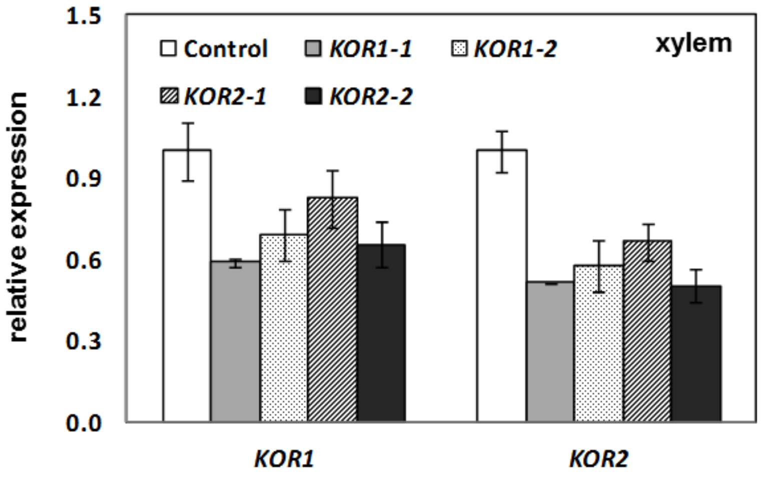

(B)

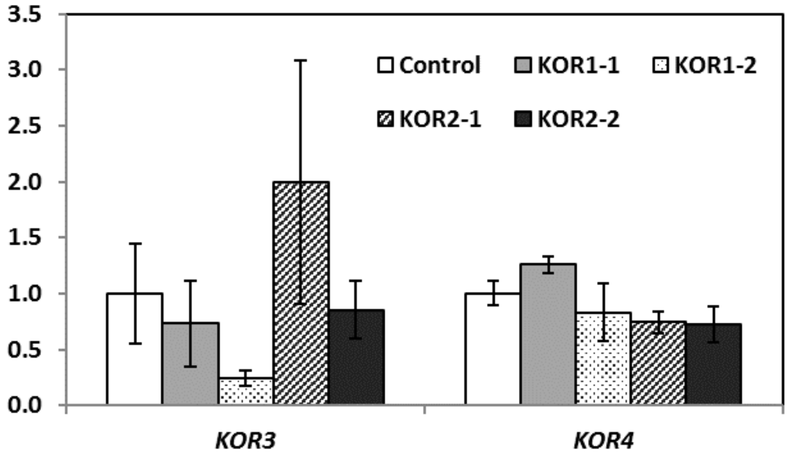
3.5
3.0
2.5
2.0
1.5
1.0
0.5
0.0
Control
KOR1-1
KOR1-2
KOR2-1
KOR2-2
KOR3
KOR4

(C)

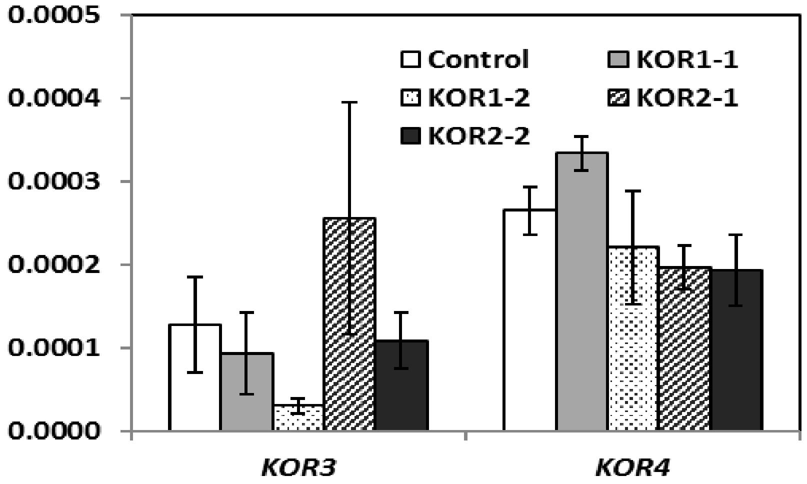
0.0005
0.0004
0.0003
0.0002
0.0001
0.0000
Control
KOR1-1
KOR1-2
KOR2-1
KOR2-2
KOR3
KOR4

Figure S5. Cell wall composition of control and *PdKOR* downregulated lines (downregulated in both *PdKOR1* and *PdKOR2*). (A) percentage (%) cellulose content, (B) NMR-based cellulose crystallinity, (C) degree of polymerization of cellulose, (D) lignin content and (E) S/G (syringyl-to-guaiacyl) ratio are represented as means±SE (n=3-5). (A-E) represent values from debarked stem samples. * indicates statistically significant, $p \leq 0.05$ based on Student's *t*-tests.

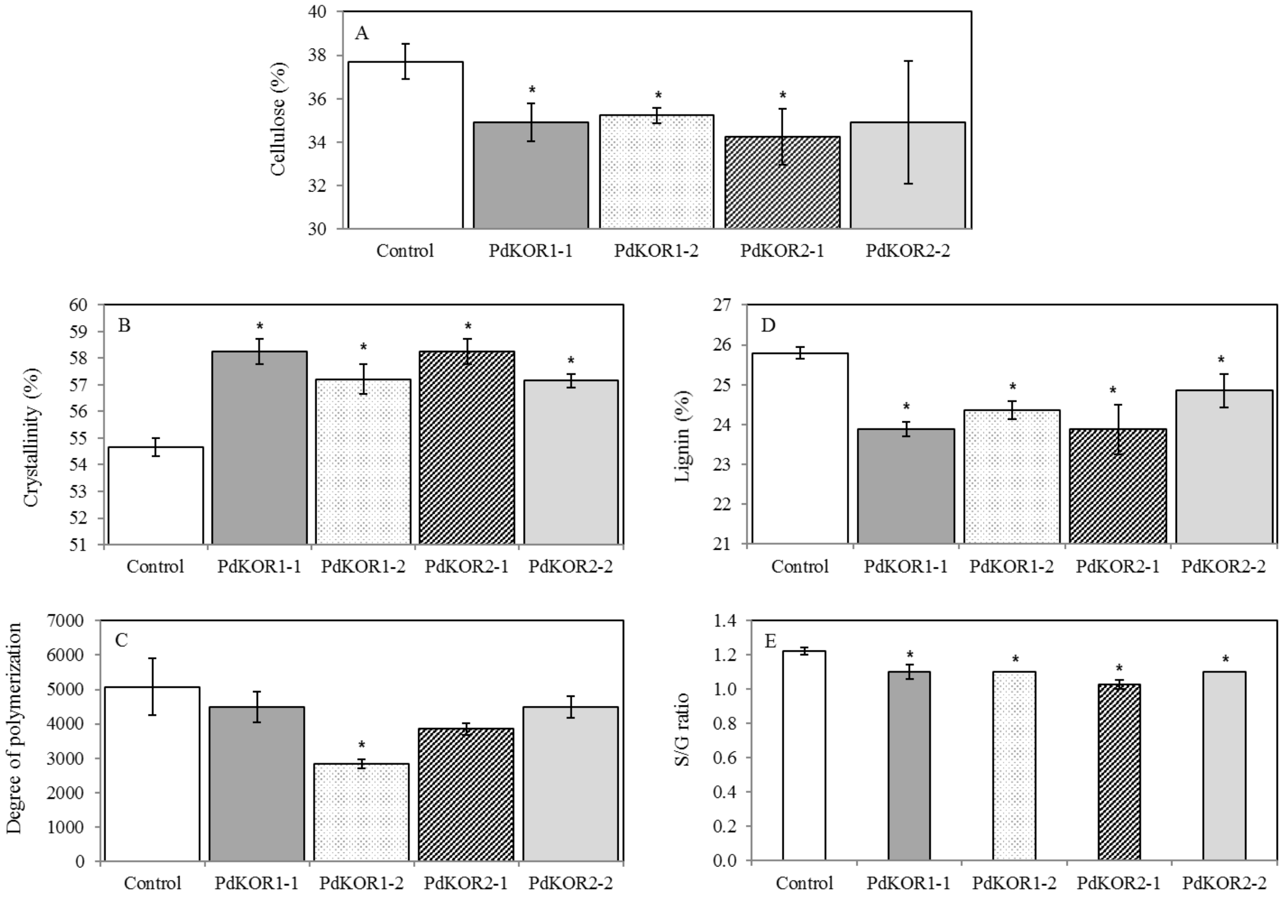

Supplement: Supplementary file 1 [file Data_Sheet_1.PDF]
